# Targeting the spatial context of obesity determinants via multiscale geographically weighted regression

**DOI:** 10.1186/s12942-020-00204-6

**Published:** 2020-04-05

**Authors:** Taylor M. Oshan, Jordan P. Smith, A. Stewart Fotheringham

**Affiliations:** 1 Center for Geospatial Information Science, Department of Geographical Sciences, University of Maryland, College Park, MD 20740 USA; 2 School of Geographical Sciences & Urban Planning, Arizona State University, Tempe, AZ 85281 USA; 3 Spatial Analysis Research Center, School of Geographical Sciences and Urban Planning, Arizona State University, Tempe, AZ 85281 USA

**Keywords:** Obesity, Spatial epidemiology, Urban health, Multiscale, GWR

## Abstract

**Background:**

Obesity rates are recognized to be at epidemic levels throughout much of the world, posing significant threats to both the health and financial security of many nations. The causes of obesity can vary but are often complex and multifactorial, and while many contributing factors can be targeted for intervention, an understanding of where these interventions are needed is necessary in order to implement effective policy. This has prompted an interest in incorporating spatial context into the analysis and modeling of obesity determinants, especially through the use of geographically weighted regression (GWR).

**Method:**

This paper provides a critical review of previous GWR models of obesogenic processes and then presents a novel application of multiscale (M)GWR using the Phoenix metropolitan area as a case study.

**Results:**

Though the MGWR model consumes more degrees of freedom than OLS, it consumes far fewer degrees of freedom than GWR, ultimately resulting in a more nuanced analysis that can incorporate spatial context but does not force every relationship to become local *a priori*. In addition, MGWR yields a lower AIC and AICc value than GWR and is also less prone to issues of multicollinearity. Consequently, MGWR is able to improve our understanding of the factors that influence obesity rates by providing determinant-specific spatial contexts.

**Conclusion:**

The results show that a mix of global and local processes are able to best model obesity rates and that MGWR provides a richer yet more parsimonious quantitative representation of obesity rate determinants compared to both GWR and ordinary least squares.

## Introduction

Obesity rates are at epidemic proportions throughout much of the world [1]. However, rates vary greatly across spatial contexts and different socio-demographic groups, taking a particularly disproportionate toll on low-income individuals and racial/ethnic minority populations [2]. Beyond individual health consequences, such as hypertension and heart disease, obesity inflates the price of healthcare—treating the array of otherwise preventable diseases and health conditions associated with obesity results in annual expenditures in the hundreds of billions of dollars [3–5]. Furthermore, the rate of obesity continues to increase despite attempts at intervention and prevention, resulting in concomitant increases in projected healthcare costs. Therefore, the global obesity epidemic poses significant threats to both the health and financial security of the population.

The causes of obesity can vary but are often interconnected and compounding. Factors beyond genetics play a central role in the likelihood of an individual becoming obese. Such factors include those primarily related to lifestyle (e.g., diet and physical activity) and social networks (e.g., media and peer pressure) [6–9], as well as other geographic considerations (e.g., urban areas vs. rural areas and accessibility) and the role of the built environment [10–14]. An enormous body of literature exists that examines the factors associated with obesity and potential approaches to mitigate the issue (e.g., [15, 16]). Many contributors to obesity can be targeted for interventions; however, an understanding of where these interventions are needed and whether or not they are successful is essential for effective policy implementation [3, 17]. This has prompted an interest in incorporating spatial context into the analysis and modeling of obesity determinants (e.g., [18–25]. In particular, geographically weighted regression (GWR) is a method that is frequently employed to understand how spatial determinants of obesity vary across space [26–40]. A drawback of GWR is that it assumes that all of the relationships being modeled vary at a single spatial scale, limiting the potential to characterize spatial context. In contrast, the recently developed multiscale (M)GWR allows multiple spatial scales to be expressed simultaneously [41], but it has not yet been applied to model obesity determinants.

Therefore, the goal of this research is to better target the spatial context of obesity determinants using an explicitly multiscale approach (i.e., MGWR). It first targets the limitations of previous efforts to capture the spatial context of obesity determinants when employing GWR and suggests several best practices for building, interpreting, and reporting results for a GWR model. Second, it provides a novel analysis that demonstrates the advantages of using MGWR to target the spatial context of obesity determinants. In particular, this study concentrates on the Phoenix, Arizona, metropolitan area, modeling how obesity determinants can vary across the urban environment and different socio-economic communities. As a result, some of the shortcomings of previous work are overcome, and a more consistent methodology is suggested to analyze the spatial context of different types of obesity determinants across different study areas. The remaining sections are organized as follows: first, limitations of previous applications of GWR to obesity determinants are highlighted; then the study area data are introduced and thr MGWR methodology is described; the results are then presented; and finally, some discussion and conclusions are provided.

## Background and previous work

At the core of GWR is a data-borrowing procedure that creates spatially local subsets of data to enable the estimation of model parameters at any number of locations in a study area. This contrasts with traditional “global” ordinary least squares (OLS) regression and spatial regression models, such as the simultaneous autoregressive model and the conditional autoregressive model (e.g., [23, 24], that estimate a single set of parameters, each of which is assumed to be constant across the entire study area. Comparing local parameter estimates across space is advantageous because it reveals whether and how the determinants of obesity vary across geographic space (i.e., spatial context); issues ignored in a global model. Thus, GWR provides a mechanism for not only exploring where a model is an effective representation but for identifying which factors contribute towards such a representation for individual locales. However, there are several limitations in existing studies that utilize GWR to extract relevant spatial contexts of obesity determinants, making it challenging to interpret their results, gain collective insight about obesogenic processes, and suggest effective policy implementations.

First, several studies rely upon a univariate GWR model (i.e., simple regression) or a series of univariate GWR models to investigate obesity determinants [31, 32, 36, 39]. However, it is generally acknowledged that the causes of obesity are complex and multifactorial. Therefore, a more appropriate way to represent obesogenic processes is through multivariate models (i.e., multiple regression) that can simultaneously account for several conditional relationships. This also implies that the spatial patterns identified for each obesity determinant in a multivariate GWR model are dependent upon the other included variables. As a result, the spatial patterns identified by any univariate GWR model can only be considered in isolation, are not likely robust when other factors are considered, and are more susceptible to omitted variable bias.

Second, there is the issue of potential multicollinearity in local statistical models. While [42] demonstrate that GWR is robust to the malignant effects of multicollinearity when the sample size is large, it is still prudent to check for local multicollinearity that may not be detected by traditional global measures. Several tools are available for diagnosing multicollinearity amongst the local subsets of data created during a GWR or MGWR calibration [43–45]. Unfortunately, in the existing literature, there are many obesity applications of GWR that do not examine potential multicollinearity at all [30, 31, 33, 35] or only consider it using global diagnostics [27–29, 36, 38, 40]. This is problematic because extreme (global or local) multicollinearity can cause parameter estimate instability, unintuitive parameter signs, high $R^{2}$ diagnostics despite few or no significant parameters, and inflated standard errors of the parameter estimates [46], complicating the interpretation of process heterogeneity and spatial context. Even more concerning, in some cases, the presence of multicollinearity prompted researchers to rely upon univariate GWR models rather than comprehensive multivariate regression models of obesity [32, 36], an issue already highlighted above.

The third drawback with existing research employing GWR to study obesity that causes reservation is that several previous applications report a relatively low level of explanatory power (i.e, $R^{2}$) [28, 29, 34–37, 39]. Studies that report low global explanatory power, sometimes accounting for less than 10% of the variation in the dependent variable, are not likely capturing robust relationships. The use of GWR in some obesity studies furnished substantially higher model fit metrics over an analogous global model; however, these models still had relatively low explanatory power. GWR cannot remedy a poorly defined model, and it could be that some of the increased model fit is due to overfitting to the data. Additionally, some studies fail to report any model fit criteria, making it difficult to assess the explanatory power of the results and build upon them [31–33].

A more general lack of reporting is the fourth issue found in previous obesity applications of GWR. For example, some studies present GWR results without reporting any output for an analogous global model [35, 37]. As mentioned above, it is paramount to first find a robust global model before moving onto a local model and in these cases, it is unclear whether or not this step was undertaken. Furthermore, the results from a global model are useful for interpreting the results from a GWR in order to indicate which local relationships deviate from the global model and in which way. Another example is that several studies are either vague or do not report the choice of kernel function employed within their GWR analysis [31, 35, 37, 40]. This can have implications for how the bandwidth is interpreted as an indicator of process scale, as well as limit the ability to replicate or reproduce a methodology. Moreover, the bandwidth is also frequently not reported [26, 28, 29, 31–33, 36, 37, 39, 40], leaving valuable insights regarding process scale and spatial context untapped.

Fifth, many of the previous obesity studies using GWR do not consider the uncertainty of the local parameter estimates (i.e., hypothesis evaluation using a *t*-test) [28, 29, 31, 32, 34, 38, 39]. It is imperative to consider parameter estimate uncertainty to ensure that estimates that are not statistically different from zero are not meaningfully interpreted. Even in the cases where prior obesity studies do apply local hypothesis testing, they do not account for the fact that multiple dependent hypothesis tests are being carried out by applying the proposed GWR-specific test correction of [47]. The result is that the overall hypothesis testing framework employed in previous research is not conservative enough and could lead to mistakenly identifying spatial patterns. Consequently, previous results should be interpreted cautiously.

A final limitation of these previous studies is that they do not employ the recently developed multiscale extension of GWR (MGWR). This means that in their studies, there was an implicit assumption that each obesity determinant operated at the same spatial scale (i.e., the same kernel bandwidth for each variable). However, it is much more likely that the complex social, economic, and demographic factors associated with obesity may each vary at different scales (i.e., unique kernel bandwidths for each variable). For example, ethnicity can differ sharply across metropolitan areas (i.e., segregation) and as discussed above, certain ethnicities are more susceptible to higher obesity rates. In contrast, people are all subject to the effects of aging, and it could be that the relationship between age and obesity rates is independent of spatial context when other factors are taken into consideration. When it is assumed that the same spatial scale applies to both of these relationships, it is possible that the true patterns across space are obfuscated because the model is misspecified. As a result, it is important to utilize a multiscale approach, such as MGWR, in order to more accurately reveal the spatial context of complex spatial processes. An example using MGWR to model local determinants of obesity is detailed below and several suggestions are made to alleviate some of the challenges outlined previously.

## Methodology

### Study area

**Fig. 1:**
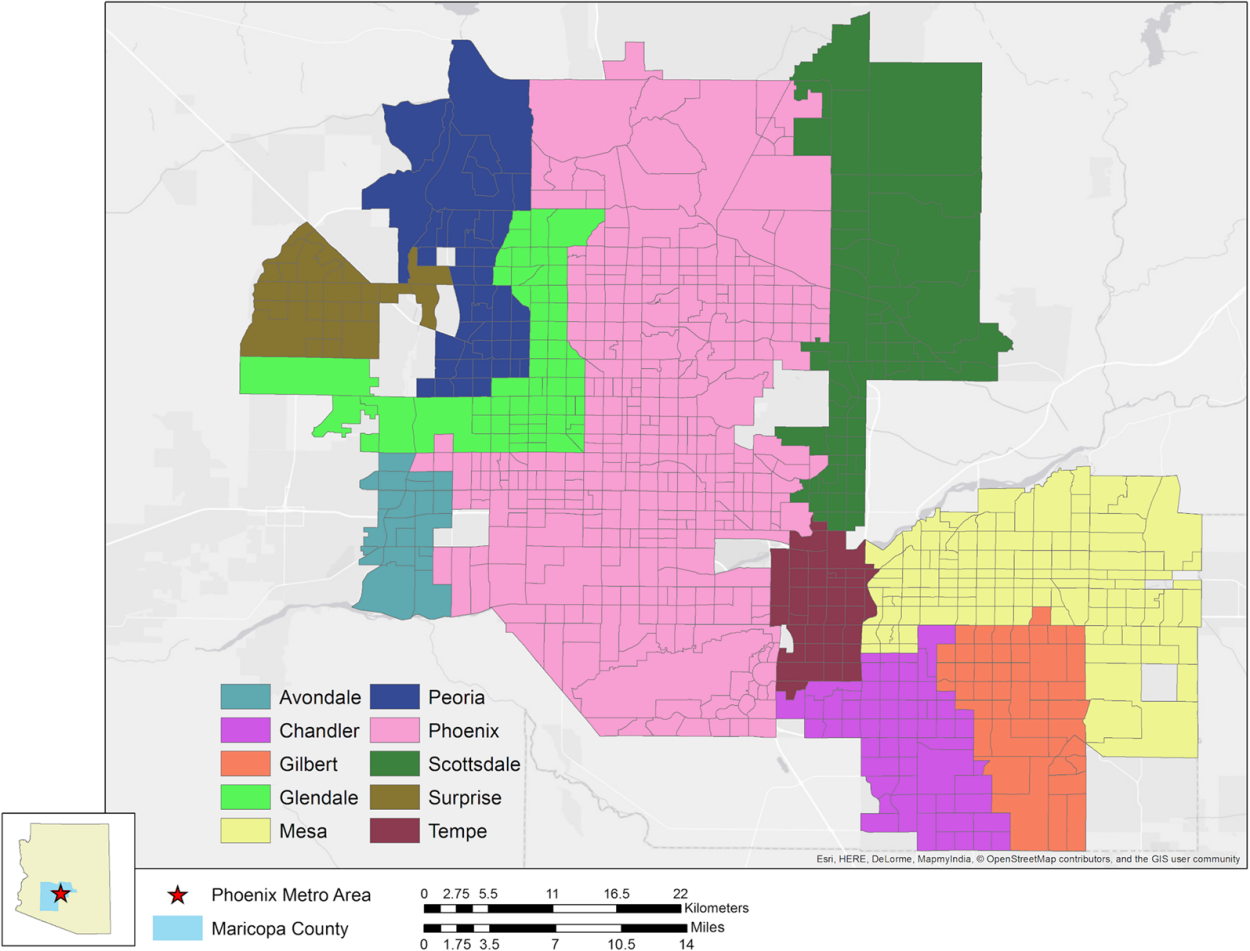
The Phoenix metropolitan area as covered in the 500 Cities Project. Obesity rate data is available for 10 individual cities

In the state of Arizona, 28.9% of adults are considered obese, which is below the United States national average [48]; however, when delineated along sociodemographic characteristics, this number varies dramatically and is concordant with national trends [49]. Through targeted surveying and other statistical analyses [50], the “500 Cities Project”^1^ developed by the Centers for Disease Control (CDC) and the Robert Wood Johnson Foundation was able to develop a representative profile at the census tract level for cities across the nation [51]. Of the 12 cities evaluated within the state of Arizona, 10 of them are located within the Phoenix metropolitan area (Fig. 1). The city of Phoenix and its surrounding communities constitute one of the largest urban complexes in the US, both in terms of land area and population, encompassing approximately 3000 km^2^ and a population of over 4 million individuals. The expansive and rapid nature of development in the metropolis has resulted in a sprawling, demographically diverse landscape [52] which makes it an ideal candidate for studying the spatial influence of an array of factors related to adult obesity across a large urban area.

**Fig. 2:**
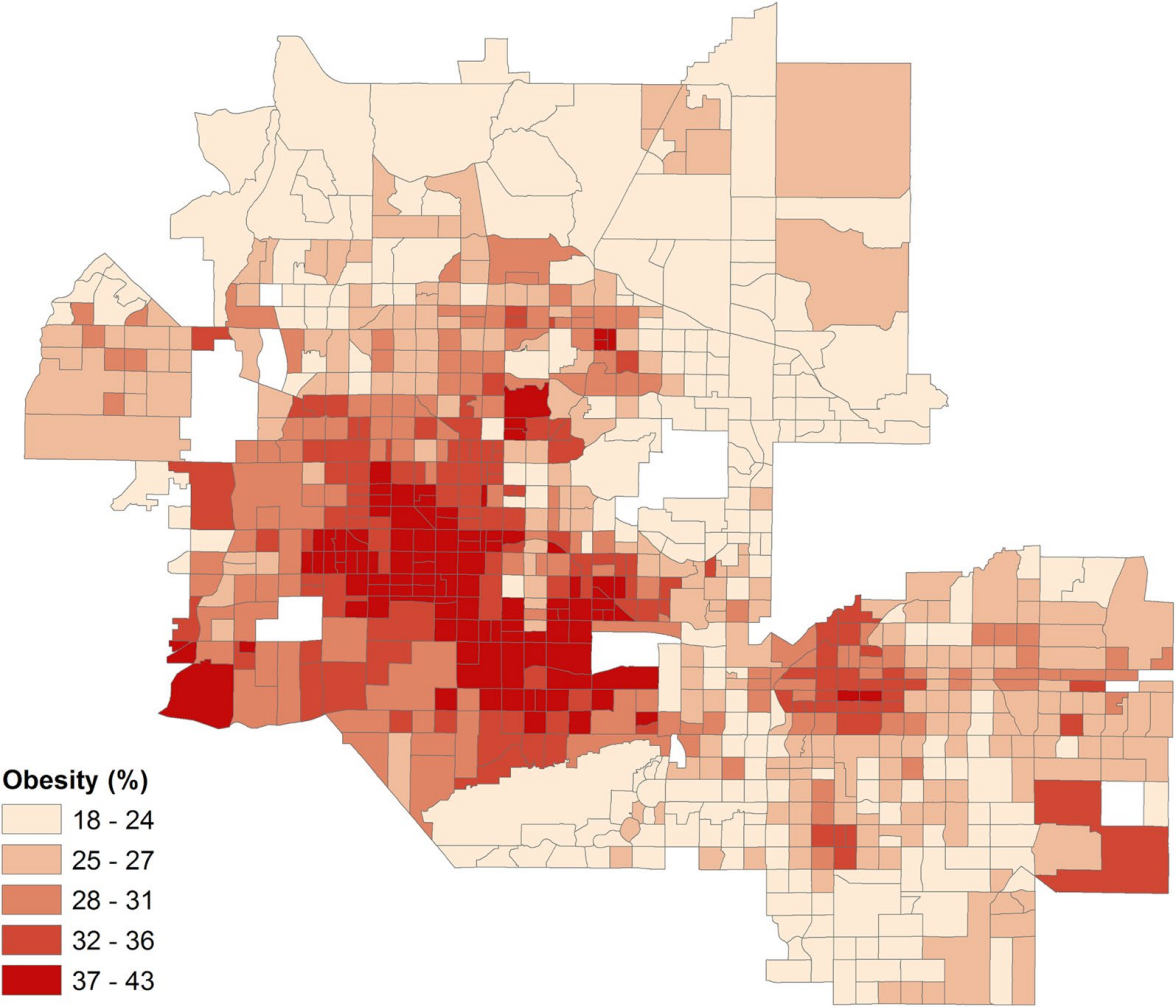
Percentage of obese population by census tract in the Phoenix metropolitan area

In the Phoenix metropolitan area, the percentage of adults who are obese per Census tract varies greatly depending upon location (Fig. 2). The highest incidences of obesity, in excess of 40%, are found within the urban core of the city of Phoenix. This region is notable because it has significant concentrations of low-income and racial/ethnic minority households, which are often more susceptible to obesity. Throughout much of the metropolitan area, though, the obesity rate remains moderate-to-high. Even within wealthier suburban communities on the fringe, some tracts report up to 25% of the population as obese. Hence, it is likely that contributors to obesity within the Phoenix metropolitan area may include additional factors other than income and ethnicity.

### Data

All of the variables used in the analysis were joined to census tract shapefiles generated by the US Census Bureau. Obesity rates were reported by the 500 Cities Project as an estimated percentage of adults within a census tract with a body mass index of 30.0 kg/m^2^ or greater for the year 2014 and can be downloaded directly from the project website.^2^ In total, there were 815 suitable tracts identified within the Phoenix metropolitan area.^3^ A variety of different explanatory variables were selectively chosen to develop a robust model (Table 1), which are explained below.

**Table 1:** Description of the variables included in the study

Outcome variable
Obesity^a^: Percentage of the population with a body mass index ≥ 30.0 kg/m^2^
Explanatory variables
Annual Checkup^a^: Percent of the population receiving an annual checkup in past year
African American^b^: Percent of individuals identifying as African American
Hispanic^b^: Percent of individuals identifying as Hispanic/Latino, independent of race
SNAP^b^: Percent of households on Supplemental Nutrition Assistance Program
College^b^: Percent of the population with a bachelor’s degree or higher
Food Desert^c^: Share of low-income population beyond 1 mile from a supermarket
Mean NDVI^d^: Averaged Normalized Difference Vegetation Index for Census tract

^a^500 Cities Project, 2014

^b^ACS, 2015

^c^FDA, 2015

^d^NAIP, 2010

The data generated by the 500 Cities Project allows for fine-scale evaluation of obesity at the metropolitan level for census tracts by downsampling from counties using statistical techniques [53–56]. Data from the project fall into three general categories: health outcomes, prevention, and unhealthy behaviors. Initially, a large number of potential variables were drawn from the project dataset, including those associated with unhealthy behaviors, such as smoking, drinking, lack of sleep, physical activity, and health insurance. However, these variables exhibited high collinearity based on their global variance inflation factors (VIFs) when evaluated against one another (i.e., greater than 10) or pose the issue of endogeneity. Collinearity occurs when the variables represent redundant information. For example, those who frequently smoke and drink often do so in tandem [57], and if both behaviors are conducive to obesity than it is not possible to decipher the individual relationships between these variables. Endogeneity occurs when relationships are circular and it is not possible to identify a potential direction of causality. For example, the physical activity variable was not included in the model because it is not clear whether or not lower physical activity causes higher obesity rates or if higher obesity rates cause individuals to engage in less physical activity [58, 59]. Ultimately, the percentage of individuals who reported undergoing annual checkups was the only explanatory variable from the 500 Cities Project included in the study due to the issues described above. This variable is defined as the number of individuals that received at least one routine doctor visit, such as an annual physical examination, and does not include visits for specific ailments. Primary care is frequently cited as a means of reducing the likelihood of whether or not an individual will be susceptible to obesity and the negative health conditions associated with it [49, 60–63].

Several additional explanatory variables were obtained from the 2011–2015 5-Year American Community Survey (ACS). These include median tract age, the percentage of African American and Hispanic populations, percent participation in the Supplemental Nutrition Assistance Program (SNAP), percentage of college degree attainment, and average household income. Age has been shown to be a key determinant of obesity, with middle-aged adults having a far greater likelihood of being obese compared to older-aged and younger-aged adult groups [2, 64, 65]. Race and ethnicity are frequently regarded as reliable predictors of obesity where minority populations are considered to be especially vulnerable to higher rates of obesity [8, 49, 66, 67]. The percentage of households receiving SNAP benefits (formerly known as food stamps) was included because there is vigorous debate as to whether the program increases or decreases obesity rates in low-income communities [49, 68–71]. Lastly, the percentage of the tract population with at least a bachelor’s degree was included, as there is evidence that individuals with at least some college education are less likely to be obese [65, 72, 73]. Average household income and median age were ultimately omitted from the final model due to collinearity, likely because low-income is a requirement for SNAP enrollment and middle- and older-aged populations are more likely to receive routine medical attention.

A variable representing food deserts was also incorporated into the study because food access is frequently cited as a contributor to poor dietary behavior [30, 49, 74]. While the definition of food deserts typically varies from study to study [75], a widely deployed measure is the proportion of low-income individuals residing within one mile of a supermarket in an urban census tract as denoted by the US Department of Agriculture (USDA) [49, 76]. A limitation of this definition is that only grocery stores that produce more than $2,000,000 in annual sales per year are considered as food retailers [77]. Due to this high revenue threshold, the contribution of many smaller food retailers such as bodegas or other local vendors is not effectively captured using this measure [78, 79]. Additionally, the USDA metric assumes a static population that does not consider potential mobility between tracts [79–81]. Despite these limitations, the USDA definition of a food desert is still widely used as a metric of food access in many studies [82] and was therefore obtained^4^ and employed here.

The last variable considered in this study was vegetative cover as a proxy for the presence of greenspace in a neighborhood. Greenspace availability is frequently cited as having a positive impact on residents’ health, and by extension on obesity, because they provide cool places for recreation, especially in large metropolitan areas that are susceptible to the Urban Heat Island effect [21, 83, 84]. In desert-scape cities like Phoenix, greenery can also indicate socioeconomic class, as wealthier Caucasian communities frequently have more access to such features (be it in the form of parks, walking paths, or even landscaped yards) than poorer minority communities [85, 86]. To incorporate a proxy for green cover into the study, the Normalized Difference Vegetation Index (NDVI) was derived from 1-m National Agriculture Imagery Program^5^ (NAIP) imagery generated in June 2010. NDVI is a unitless measure of vegetation per image pixel such that vegetated features will yield high values and non-vegetated features, such as rock and pavement, will produce low values [87]. NDVI values were averaged across the pixels located within each census tract employed in the study.

### Geographically weighted regression and multiscale extensions

Conventional “global” regression modeling assumes relationships are constant across a study area and can be characterized by:

1 $$ y\left( i \right) = \hat{\beta }_{0} \left( i \right) + \hat{\beta }_{1} X_{1} \left( i \right) + \hat{\beta }_{2} X_{2} \left( i \right) + \cdots + \hat{\beta }_{k} X_{k} \left( i \right) + \varepsilon \left( i \right) $$

where $y\left( i \right)$ is the observation of the dependent variable at $ith$ location, $\hat{\beta }_{0}$ is the estimated intercept, $X_{k} \left( i \right)$ is the observation of the $kth$ explanatory variable at the $ith$ location, $\hat{\beta }_{k}$ is the $kth$ parameter estimate, and $\varepsilon \left( i \right)$ is a random error term for $i = \left\{ {1, 2, 3, \ldots , n} \right\}$. In reality, however, many spatial processes vary with geographic context, in which case the above specification is misspecified because it assumes the values of the parameter estimates are constant and apply to every location within the study area. GWR relaxes the assumption of spatial stationarity associated with global models and allows relationships to vary from location to location [88]. In essence, GWR explicitly incorporates geographic context by allowing parameter estimates to be derived for each location of interest, which is denoted by:

2 $$ y\left( i \right) = \hat{\beta }_{0\left( i \right)} + \hat{\beta }_{1\left( i \right)} X_{1} \left( i \right) + \hat{\beta }_{2\left( i \right)} X_{2} \left( i \right) + \cdots + \hat{\beta }_{k\left( i \right)} X_{k} \left( i \right) + \varepsilon \left( i \right) $$

where the parameter estimates are now also indexed by the $ith$ location. Parameter estimates are obtained at each location by calibrating a locally weighted regression using the following estimator in matrix form:

3 $$ \hat{\varvec{\beta }}\left( \varvec{i} \right) = (\varvec{X^{\prime}W}\left( i \right)\varvec{X})^{ - 1} \varvec{X^{\prime}W}\left( i \right)\varvec{y} $$

where $\hat{\beta }\left( i \right)$ is an $k \times 1$ vector of parameter estimates, $\varvec{X}$ is an $n \times k$ matrix of explanatory variables, $\varvec{y}$ is a $k \times 1$ vector of observations for the dependent variable, and $\varvec{W}\left( i \right)$ is a spatial weights matrix that encodes a data-borrowing scheme designed to allow data points closer to location $i$ to have a stronger influence on the local regression.

The weighting matrix, $\varvec{W}\left( i \right)$, is characterized by a kernel function, a measure of proximity, and a bandwidth parameter that controls the intensity of weighting or data-borrowing (i.e., scale). A popular choice in previous GWR models of obesity is a Gaussian kernel function and Euclidean distance-based measure of proximity [28, 32, 36, 38, 39]. However, in this study, a bi-square kernel function with a nearest-neighbor measure of proximity is employed. This data-borrowing scheme is ideal for two reasons. First, nearest-neighbor definitions of proximity are more robust to irregular spatial sampling. Second, the bi-square kernel function has the interpretation that the bandwidth is the number of nearest-neighbors at which the data is weighted to exactly zero and further observations have no influence on each local regression [43]. This is useful for comparing bandwidths and interpreting them as indicators of spatial scale. An optimal bandwidth parameter is selected by minimizing a corrected Akaike information criterion (AICc), which provides a balance between model variance and bias [88, 89].

Since GWR produces sets of local parameter estimates using overlapping subsets of data, it is necessary to account for multiple hypothesis tests that will not be independent. Whereas a *t*-value larger than ± 1.96 for larger sample sizes indicates an estimate is different from zero at the 95% confidence level (1 - $\alpha ; \alpha = 0.05$) in a global model, a more conservative (i.e., smaller) $\alpha$-value is needed to maintain the 95% confidence level in GWR. Therefore, a GWR-specific correction is applied to obtain to the $\alpha$-value such that:

4 $$ \alpha = \frac{{\xi_{{}} }}{{\frac{ENP}{p}}} $$

where $\xi_{{}}$ is the desired joint type I error rate (i.e., 0.05), $ENP$ is the effective number of parameters in GWR that depends upon the data-borrowing scheme, and $p$ is the number of explanatory variables in a given model. The ratio $\frac{ENP}{p}$ ($ENP > p$) is representative of the number of multiple tests for a given data-borrowing scheme. If $ENP = p$ then $\xi_{ } = \alpha$ and the tests performed by GWR and a global regression are equivalent. Using Eq. (4) to obtain an adjusted $\alpha$ it is possible to derive a corrected critical *t*-value that is likely larger than ± 1.96 and is, therefore, more conservative [47]. Previous GWR models of obesity determinants did not include this hypothesis testing framework, which means it is possible that some parameter estimates may have been mistakenly deemed statistically non-zero.

One limitation of the GWR framework described above is that the same bandwidth is assumed to apply for each relationship in the model, which means the data are weighted at the same spatial scale. A recent extension to the GWR framework [41] overcomes this limitation by reformulating GWR as a generalized additive model (GAM):

5 $$ \varvec{y}_{\varvec{i}} = \mathop \sum \limits_{j = 1}^{k} f_{ji} + \varepsilon_{i} $$

where $f_{ji}$ is a smoothing function (i.e., spatial weight or data-borrowing scheme) applied to the $jth$ explanatory variable at location $i$. Then, it is possible to calibrate the model using a backfitting algorithm that derives a set of bandwidth parameters for the $j$ processes being modeled. Since each bandwidth represents a unique scale for each process, this extension is known as multiscale (M)GWR. A major advantage of MGWR is that it can more accurately capture the spatial heterogeneity within and across spatial processes, minimize overfitting, mitigate concurvity (i.e., collinearity due to similar functional transformations), and reduce bias in the parameter estimates [41, 43, 89, 90].

Another benefit of using MGWR over GWR is that an adjusted $\alpha$-value and critical *t*-value can be computed for each of the $j$ relationships being modeling, since they may have distinct data-borrowing schemes and differing effective numbers of parameters. As a result, hypothesis testing for the $jth$ set of parameter estimates is carried out using:

6 $$ \alpha_{j} = \frac{{\xi_{{}} }}{{ENP_{j} }} $$

where $ENP_{j}$ is the effective number of parameters for the $jth$ model term [91]. $\alpha_{j}$ can then be used to derive a covariate-specific critical *t*-value. To the knowledge of the authors, this paper provides the first application of both MGWR and its associated hypothesis testing framework for modeling obesity determinants.

To investigate obesity determinants in Phoenix, we first calibrated a global model using OLS regression, which assumes processes to be constant across the study area. Subsequently, a GWR and MGWR model were calibrated^6^ using a golden section search bandwidth selection routine to obtain optimal bandwidths. The response and explanatory variables were standardized to have a mean of zero and variance of unity so that the bandwidths from MGWR are free from the scale and variation of the explanatory variables, facilitating the relative comparison of bandwidths [41, 93]. Following [43], composite maps were prepared to visualize the parameter estimates and their uncertainty with estimates statistically indistinguishable from zero displayed in grey. The GWR and MGWR maps are presented side-by-side for each variable in order to compare the parameter estimate spatial heterogeneity across the two models. Lastly, maps of the local condition number were prepared to investigate local multicollinearity in both GWR and MGWR. The local condition number is obtained by computing the condition number on each local subset of the design matrix that is obtained by $\varvec{W}\left( i \right) \varvec{X}$ for each location, $i$.

## Results

Results from the global model (Table 2) are first summarized in order to provide context for the GWR and MGWR results.

**Table 2:** Results from the ordinary least squares regression model

N = 815 tracts	$R^{2}$ = 0.878; Condition number = 3.788		
Variable	Coefficients	*t*-value	VIF
Intercept	− 3.795e−16	− 3.06e−14	n/a
Checkup	− 0.0680	− 3.959	1.912
African American	− 0.0267	− 1.892	1.295
Hispanic	0.1376	7.052	2.470
SNAP	0.5244	23.988	3.100
College	− 0.3007	− 13.845	2.595
Food desert	0.0422	3.303	1.058
NDVI	− 0.0358	− 2.684	1.158

The global model produces a relatively high $R^{2}$ (Table 2), indicating a large portion of the variation across obesity rates can be accounted for by the selected variables in this study. Multicollinearity is moderate-to-low since the condition number of 6.47 is below 30 and the VIFs for each explanatory variable (Table 2) are all under the common threshold of 10 [46] and the more conservative threshold of 5. Based on a standard *t*-value threshold of 1.96 for a 95% confidence level, all but the percentage of African American population is statistically non-zero. This denotes that after accounting for the percentage of Hispanic population, there is no discernible additional effect associated with minority race within this dataset. The intercept is also not statistically different from zero. However, this is expected due to the standardization of the variables in the analysis, which also allows the comparison of parameter estimate magnitudes. Table 2 shows that the most influential variable is the percentage of SNAP recipients, which has a relatively strong positive relationship with obesity rates, followed by the percentage of Hispanic population and the prevalence of food deserts. In contrast, the most influential negative association is with college-level educational attainment, followed by the population receiving an annual checkup, and mean NDVI.

**Fig. 3:**
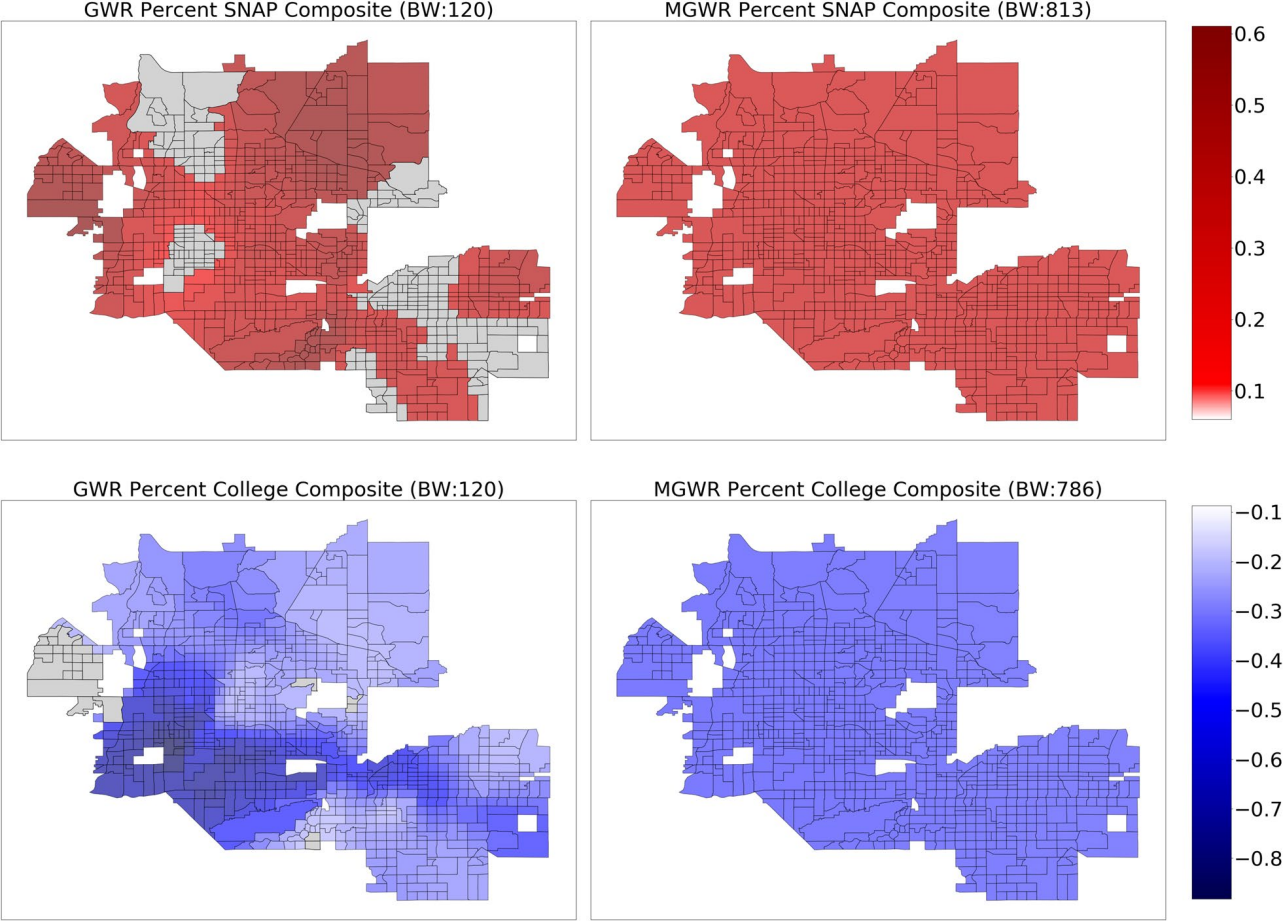
Composite maps for GWR (left) and MGWR (right) parameter estimate surfaces for percent Supplemental Nutrition Assistance Program (SNAP) (top), and percent college (bottom), which tend to show global patterns of spatial heterogeneity. Grey tracts are not statistically different from zero

**Fig. 4:**
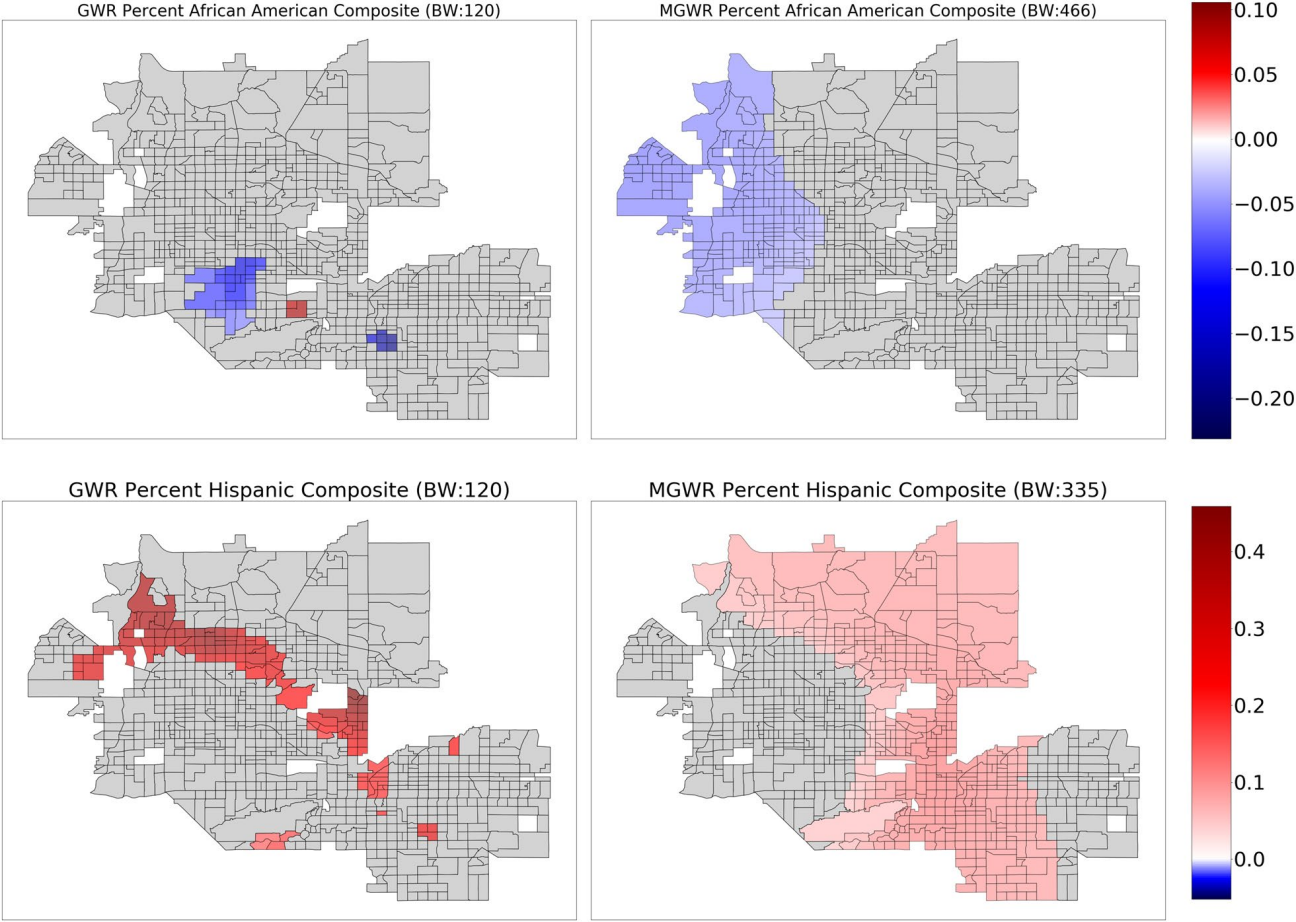
Composite maps for GWR (left) and MGWR (right) parameter estimate surfaces for percent African American (top), and percent Hispanic (bottom), which tend to show regional patterns of spatial heterogeneity. Grey tracts are not statistically different from zero

**Fig. 5:**
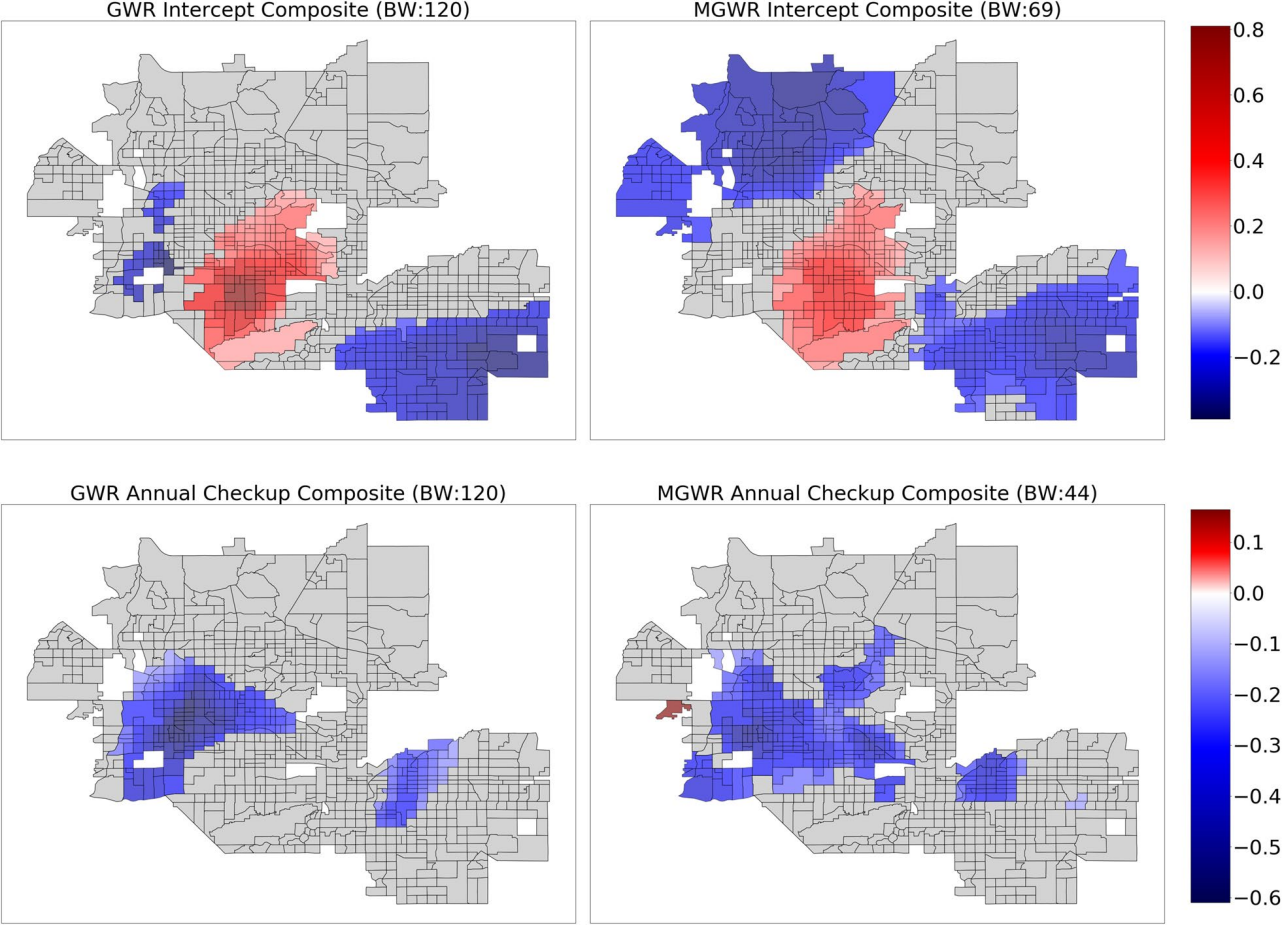
Composite maps for GWR (left) and MGWR (right) parameter estimate surfaces for the intercept (top), and annual checkup (bottom), which tend to show local patterns of spatial heterogeneity. Grey tracts are not statistically different from zero

**Fig. 6:**
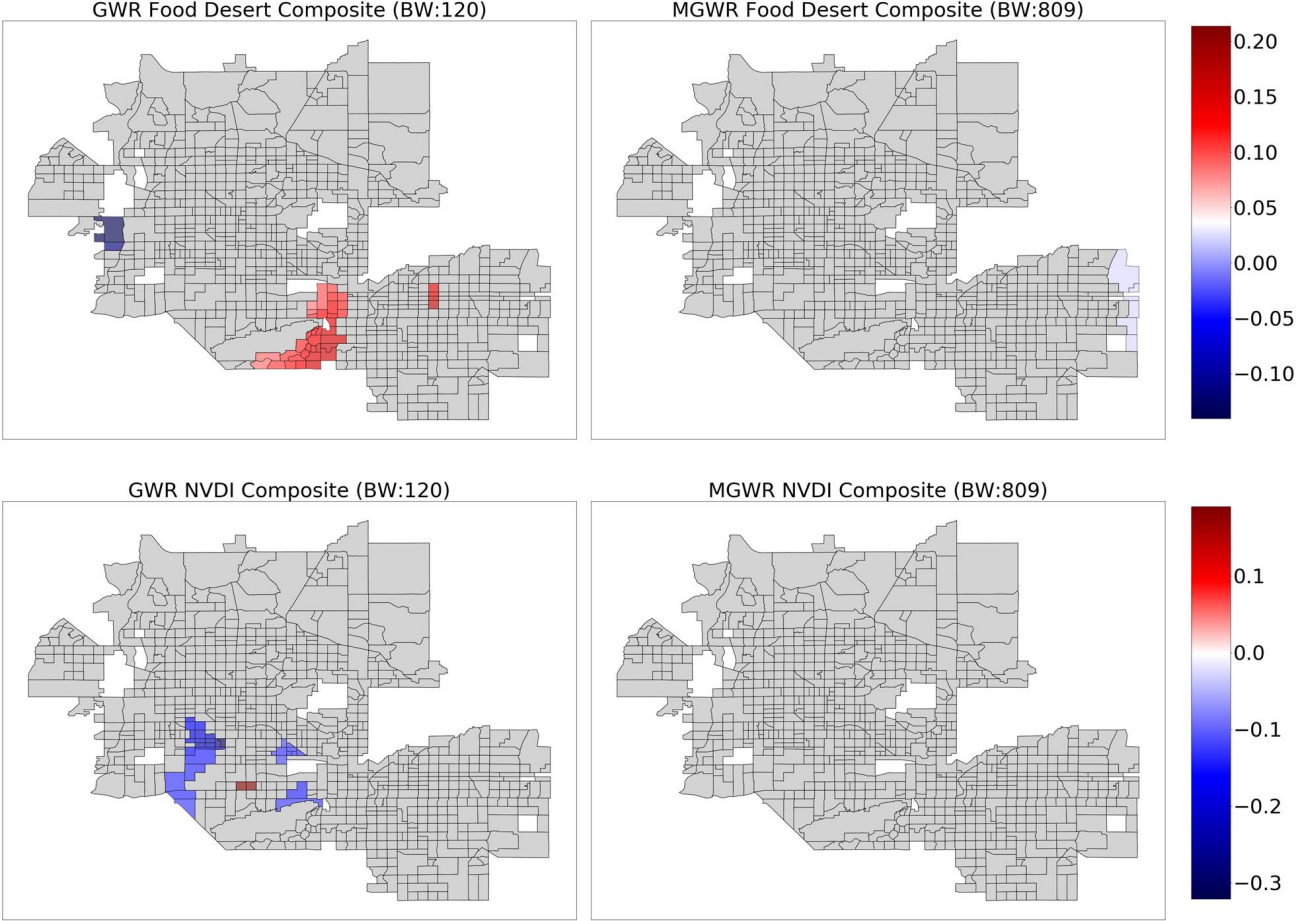
Composite maps for GWR (left) and MGWR (right) parameter estimate surfaces for food desert (top), and mean normalized difference vegetation index (NDVI) (bottom), which show no distinct patterns. Grey tracts are not statistically different from zero

**Fig. 7:**
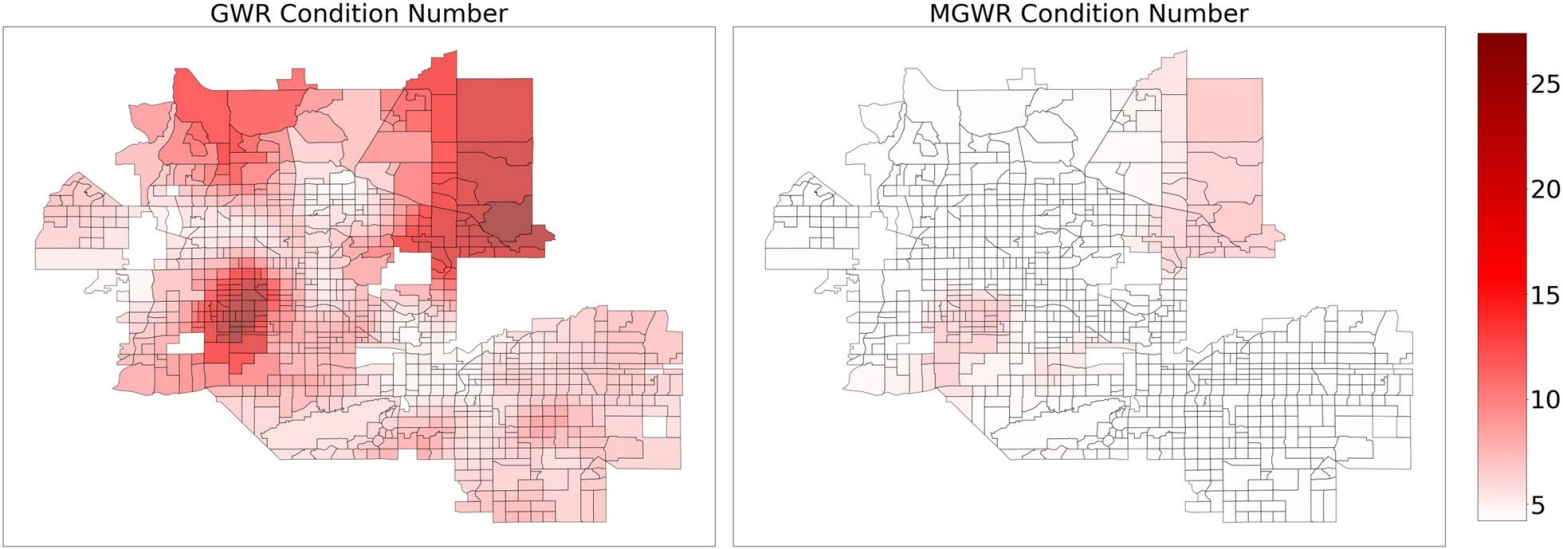
Maps of local condition numbers for GWR (left) and MGWR (right)

The above results assume that the relationships are stationary (i.e., constant) across the study area. In order to relax this assumption, GWR was applied to the same set of explanatory variables use in the global model, resulting in a relatively local optimal bandwidth of 120 nearest neighbors. The $R^{2}$ increased to 0.937 in the GWR model from 0.876 in the global model and the AIC decreased to 307.6 in the GWR model from 629.2 in the global model (Table 3). Despite these substantial increases in model fit, the parameter estimate surfaces for GWR, which are displayed in Figs. 3, 4, 5 and 6 (left) display two trends that make it difficult to interpret the results. The first trend is that several of the surfaces display a high level of spatial heterogeneity that cannot be explained. Second, some of the surfaces are almost entirely indistinguishable from a null effect aside from a few isolated tracts and are challenging to put into context. These two trends are likely due to a combination of local multicollinearity and concurvity in the local subsets of the data [42, 45, 94]. The former can be measured using local condition numbers, which are mapped in Fig. 7 (left), showing that, in some locations, multicollinearity is higher in the GWR model than in the global model. In a few cases, the condition number rule-of-thumb of 30 is approached, signaling that multicollinearity may be problematic. The latter is due to the fact that all of the processes are assumed to vary at a single scale so that the explanatory variables are all transformed using the same relatively local spatial weighting function (i.e., a bandwidth of 120). This also means that a single corrected *t*-value threshold of ± 2.950 is applied to all of the parameter estimate surfaces and could be over- or under-conservative for any of the individual surfaces [89].Therefore, it is necessary to employ MGWR to allow processes to vary at potentially unique scales.

**Table 3:** Model fit metrics for ordinary least squares (OLS) regression, geographically weighted regression (GWR), and multiscale geographically weighted regression (MGWR)

	OLS	GWR	MGWR
$R^{2}$	0.876	0.937	0.933
AIC	629.2	307.6	274.4
AICc	n/a	351.0	294.0

Calibrating a MGWR model produces a vector of optimal bandwidths that describe the spatial scale at which each process in the model varies. In Table 4, the bandwidths pertaining to each explanatory variable are listed and compared to the single bandwidth of 120 nearest neighbors resulting from GWR and the theoretical bandwidth of infinity assumed in the global model. One way to interpret these results is as follows: four relationships occur at an effectively global scale (SNAP, educational attainment, food desert, and mean NDVI) with bandwidths indicating almost all the data is included in each local subset; two processes seem to occur at a regional scale (percent African-American and percent Hispanic) with bandwidths implying several hundred nearest neighbors; and two processes vary locally (the intercept and percent annual checkup), yielding relatively small bandwidths. However, inference in MGWR can also be decomposed by process, producing individual values of the effective number of parameters and corrected *t*-values used as a threshold for hypothesis testing for each surface (Table 4). Some of the *t*-values from MGWR are larger than the corrected *t*-value from GWR of 2.95 (i.e., more conservative), while others are smaller (i.e., less conservative).

**Table 4:** A comparison of bandwidths, effective number of parameters, and critical *t*-values for ordinary least squares (OLS) regression, geographically weighted regression (GWR), and multiscale geographically weighted regression (MGWR)

	Bandwidth			Effective # Params			Critical *t*-value		
	OLS	GWR	MGWR	OLS	GWR	MGWR	OLS	GWR	MGWR
Model	n/a	n/a	n/a	8	121	83.05	1.96	2.95	n/a
Intercept	∞	120	69	n/a	n/a	27.89	n/a	n/a	3.13
Checkup	∞	120	44	n/a	n/a	43.97	n/a	n/a	3.27
Afri. Am.	∞	120	455	n/a	n/a	2.68	n/a	n/a	2.36
Hispanic	∞	120	335	n/a	n/a	3.3	n/a	n/a	2.43
SNAP	∞	120	813	n/a	n/a	1.13	n/a	n/a	2.01
College	∞	120	786	n/a	n/a	1.26	n/a	n/a	2.06
Food Des.	∞	120	809	n/a	n/a	1.38	n/a	n/a	2.10
NDVI	∞	120	809	n/a	n/a	1.44	n/a	n/a	2.11

Several further patterns are apparent upon inspection of the MGWR parameter estimate surfaces along with their uncertainty (Figs. 3, 4, 5 and 6, right) in comparison to those from GWR (Figs. 3, 4, 5 and 6, left). Based on visual patterns rather than solely on their bandwidths, the surfaces can be grouped into four categories. The first category (Fig. 3) consists of those surfaces that are effectively global and statistically non-zero (SNAP, and college education). Compared to GWR, these surfaces display little-to-no spatial heterogeneity. In concordance with the global model results, SNAP has a positive association with obesity rates across the study area while college educational attainment has a negative association with obesity rates across the study area. The second category (Fig. 4) consists of those surfaces that have a moderate number of statistically non-zero parameter estimates but still do not display spatial variation (i.e., regional pattern) (percent African American, percent Hispanic). The percentage of African American surface is clustered in a single region in the northwest corner of the study area. The characterization of this cluster is not immediately clear and requires further investigation, but is in agreement with the global model. The third category (Fig. 5) consists of those surfaces with a substantive number of statistically non-zero parameter estimates and that also display substantial levels of spatial heterogeneity (i.e., local pattern) (intercept, and annual checkup). The last category (Fig. 6) consists of mean NDVI and prevalence of food deserts have little-to-no statistically non-zero parameter estimates despite being significant in the global model.

The patterns present in the percent Hispanic, intercept, and annual checkup variables require some additional contextualization in order to interpret them. Hispanic ethnicity shows a constant (i.e., no spatial heterogeneity) positive association with obesity rates in a large portion of North Phoenix, Peoria, Scottsdale, and Tempe. Though other areas, such as downtown Phoenix, Central Phoenix, and South Phoenix also have high Hispanic populations, they do not have a statistically robust association with obesity rates. This could be due to noise and uncertainty in the data [95] or because other variables in the model are accounting for the obesity rate variation. For example, the intercept and the annual checkup parameter estimates tend to be significant in areas where the Hispanic population variable is not. This trend could also be due to areas of relatively little variation in an explanatory variable.

Parameter estimates for annual checkup tend to have a negative association with obesity rates in regions that align reasonably well with middle-aged to older communities, such as parts of Glendale, Phoenix, Peoria, and Avondale, as well as a portion of Mesa to the East. There also appears to be an outlier with a positive association in the Westernmost part study area that needs to be further investigated.

Finally, the intercept manifests in a core and periphery pattern where the core is positively associated with obesity rates and the periphery to the Northwest and Southeast are negatively associated with obesity rates. This is interesting because the intercept is not statistically different from zero in the global model due to the standardization of variables. However, in GWR and MGWR, after standardizing the variables, they are then spatially transformed at each location, so that local variable subsets no longer have a mean of zero and a non-zero parameter estimate may be obtained. Therefore, the intercept in these local models accounts for residual spatial variation after controlling for a set of given spatial factors. However, the difference here for MGWR compared to global spatial distribution modeling techniques (i.e., Gaussian process models, autoregressive models, or mixed models) is the ability to conduct local inference for the residual spatial variation separately from each of the potentially spatially varying effects for the other explanatory variables.

Overall, MGWR provides a richer yet more parsimonious quantitative representation of obesity rate determinants compared to OLS and GWR. Though the MGWR model consumes more degrees of freedom than OLS, it consumes far fewer degrees of freedom than GWR (Effective # of parameters in Table 4) and has the added benefit of being able to analyze the consumption of degrees of freedom by each model component. This ultimately results in a more nuanced analysis that can incorporate spatial context but does not force every relationship to become local *a priori*. As a result, MGWR yields a lower AIC and AICc value than GWR (Table 3), which means that MGWR provides a better model fit than GWR. At the same time, MGWR also provided a slightly lower $R^{2}$ value than GWR (Table 3), perhaps because GWR may be overfitting to the data. MGWR is also less prone to issues of multicollinearity and concurvity, which can be seen in the significantly lower local condition numbers compared to GWR (Fig. 7), which are all well below the rule-of-thumb of 30.

## Discussion

This study demonstrates the potential of MGWR to improve our understanding of the factors that influence obesity rates. While the global model performed well, its results lack spatial context. Furthermore, it was difficult to interpret the revealed spatial context of an analogous GWR model and evidence suggested that multicollinearity and concurvity may be problematic for GWR modeling of obesity due to its complex and multifactorial nature. MGWR was able to overcome these limitations, increase model fit, provide a more parsimonious model, and produce more nuanced results that include determinant-specific spatial contexts for analyzing obesity rates.

Multiscale methods, such as MGWR, may also be useful for facilitating the development of more specific policy development by framing obesity determinants through a potential mix of global, regional, and local spatial contexts. Regardless of location, (i.e., global scale), the MGWR results support the trend that neighborhoods with higher participation in SNAP are associated with higher rates of obesity while living in a neighborhood with higher rates of college-level educational attainment is linked with lower obesity rates. These determinants may, therefore, be ideal to focus on if the goal of a policy is to have a broad impact across a study area. In contrast, populations that receive annual checkups and neighborhoods with higher minority populations (i.e., Hispanic) have more regional and local relationships with obesity rates based on MGWR in this study. The former is associated with lower obesity rates and the latter is associated with higher obesity rates. These conclusions are generally in alignment with results from the global model, but spatial variation in the parameter estimate surfaces from MGWR support the possibility to target specific neighborhoods for interventions. For example, if funding or time constraints require resources to be allocated only to a limited number of neighborhoods, then it is perhaps prudent to focus on increasing accessibility to routine medical examinations in places where there is a confirmed relationship between annual checkups and obesity rates. Similarly, a policy campaign that is targeted in a region without a confirmed relationship might be evaluated for efficacy in the future to see whether or not the relationship develops over time.

Unlike the global model, the intercept in MGWR is statistically non-zero and spatial heterogeneity in the parameter estimates identifies hot spots of high and low obesity rates after controlling for the variables in the model. These spatial patterns may include both the effect of geography, as well as the effect of geographic patterning associated with omitted variables. For example, spatial context may play a distinct role in shaping human behavior (e.g., [96, 97]). Alternatively, the intercept may be useful for identifying additional determinants, policy formation, and informing follow-up investigations. For instance, on a macro scale, the overall spatial patterning of the intercept can help suggest additional spatial determinants of obesity. There is previous evidence that the built environment and remoteness may have some impact on obesity rates [34, 40] and consideration of these determinants is necessary for future multiscale analyses. On a micro scale, the urban core of Phoenix, which is associated with high levels of obesity, can be identified as a region that requires more resources. It may also prove be an ideal place to launch exploratory surveys to learn about additional non-spatial determinants within a hotspot, such as the effect of social networks [6, 7, 98].

Another interesting facet of MGWR is that it may be able to help explore the robustness of abstractions used to define explanatory variables. Both the prevalence of food deserts and mean NDVI within census tracts did not produce any statistically non-zero local associations with obesity rates. These factors are frequently discussed in the health community as central concerns for combating adult obesity. However, this research provides preliminary evidence that such effects may less prevalent in this study area or that common proxies used to represent them might not be robust when spatial context is taken into consideration. For example, aggregating 1-m pixels to census tracts or defining food access at a particular scale may produce a variable with relatively little variation. This small amount of variation would then be smoothed in MGWR even when the bandwidth is at a maximum value. In this case, all of the data points would be included in the model, but they are still weighted according to the kernel function and it could be possible that even this relatively minor amount of smoothing may obfuscate the association that existed in the global model. This suggests that generally accepted abstractions, such as the USDA’s definition of food deserts, may need to be refined depending upon the study area and the spatial units being employed.

An additional outcome of this research was a critique of previous applications of GWR for obesity modeling and a demonstration of contemporary best practices. This includes adequately reporting the chosen data-borrowing scheme, the estimated bandwidth parameter(s), and global model results, investigating local multicollinearity, considering parameter estimate uncertainty, and the use of MGWR for robust modeling of multiscale multivariate processes. Following these practices enhances interpretability of spatial context and promotes the replicability and reproducibility necessary for building generalizable theories pertaining to obesogenic processes. Through this study, the benefits of drawing conclusions regarding process scale based on determinant-specific patterns of spatial heterogeneity in parameter estimates surfaces *and* their uncertainty also became evident. Relying solely on bandwidth values to quantify scale was potentially misleading, which could be due to the fact that GWR and MGWR currently treat the bandwidth as a deterministic phenomenon [90]. Reconsidering the bandwidth as a stochastic phenomenon and incorporating uncertainty could make it possible to assess whether or not a regional bandwidth is statistically different from a global bandwidth, altering how bandwidth is interpreted as an indicator of scale.

One final note is that MGWR and the best practices suggested here may hold merit for other health outcomes, data sources, and research questions. For example, GWR has already been applied to study obesity-related behaviors [99, 100], type 2 diabetes [101], and cancers [102, 103], and it could be beneficial to extend these inquiries through the use of MGWR. Furthermore, a finer measurement scale was pursued here than is typically utilized. The 500 Cities Project distills health and behavioral data down to the census tract level, providing the potential to investigate public health issues at an unprecedented resolution. As more fine-grained sources of health data become available thanks to cheap censors and computational advancements, the potential of MGWR to resolve the spatial context(s) of a variety of health factors may become even greater.

## Conclusion

This paper provided a critical review of previous GWR models of obesogenic processes and then presented a novel application of multiscale (M)GWR to characterize the spatial context of obesity determinants using the Phoenix metropolitan area as a case study. The results show that a mix of global and local processes are able to best model obesity rates and that MGWR provided a richer yet more parsimonious quantitative representation of obesity rate determinants compared to both GWR and ordinary least squares. Best practices for building and interpreting MGWR models were suggested and contextualized policy formation strategies were discussed that may not have been available using only OLS or GWR. Moreover, it was highlighted how MGWR can potentially be used to assess the robustness of explanatory variables and the unique role the intercept can play in improving a model. Through these efforts, it was shown how to better target the spatial context of obesity determinants using MGWR.

Several avenues of future work are possible to further develop this research. First, location-specific bandwidths could be introduced in conjunction with covariate-specific bandwidths in order to further target the spatial context of obesity determinants. Second, incorporating the concept of bandwidth uncertainty may further enhance the interpretability of the spatial context(s) revealed by MGWR. Third, subsequent research can identify additional determinants to explore within MGWR models of obesity rates. Fourth, the outcome of this research could be operationalized by connecting with policy-makers to formulate, deploy, and evaluate specific obesity reduction and prevention policies. Lastly, similar MGWR model specifications can be applied in other study areas in order to validate and generalize the conclusions obtained here and to compare results for different types of urban environments. These efforts would strengthen our understanding of the multiscale spatial processes associated with obesity, increasing our ability to plan interventions, decrease health risks, and mitigate rising healthcare costs.

## Data Availability

Available upon request

## Footnotes

1 https://www.cdc.gov/500cities/.

2 https://chronicdata.cdc.gov/500-Cities/500-Cities-Census-Tract-level-Data-GIS-Friendly-Fo/5mtz-k78d.

3 Six tracts were removed from consideration due to their large size and low population.

4 https://www.ers.usda.gov/data-products/food-access-research-atlas/download-the-data/.

5 https://www.fsa.usda.gov/programs-and-services/aerial-photography/imagery-programs/naip-imagery/.

6 All GWR and MGWR results were obtained using the *mgwr* module in the Python Spatial Analysis Library (PySAL) [43], which is enhanced with several computational optimizations [92].
